# Role of Protonation States in the Stability of Molecular Dynamics Simulations of High-Resolution Membrane Protein Structures

**DOI:** 10.1021/acs.jpcb.3c07421

**Published:** 2024-03-02

**Authors:** Jonathan Lasham, Amina Djurabekova, Volker Zickermann, Janet Vonck, Vivek Sharma

**Affiliations:** † Department of Physics, University of Helsinki, 00014 Helsinki, Finland; ‡ Institute of Biochemistry II, University Hospital, Goethe University, 60590 Frankfurt am Main, Germany; § Centre for Biomolecular Magnetic Resonance, Institute for Biophysical Chemistry, Goethe University, 60438 Frankfurt am Main, Germany; ∥ Department of Structural Biology, Max Planck Institute of Biophysics, 60438 Frankfurt am Main, Germany; ⊥ HiLIFE Institute of Biotechnology, University of Helsinki, 00014 Helsinki, Finland

## Abstract

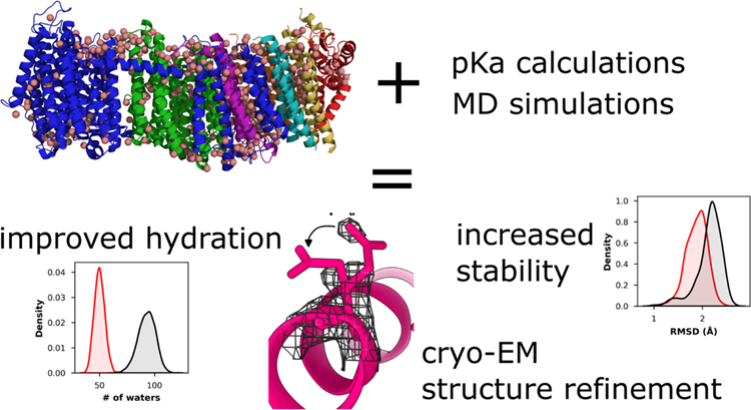

Classical molecular dynamics (MD) simulations provide unmatched spatial and time resolution of protein structure and function. However, the accuracy of MD simulations often depends on the quality of force field parameters and the time scale of sampling. Another limitation of conventional MD simulations is that the protonation states of titratable amino acid residues remain fixed during simulations, even though protonation state changes coupled to conformational dynamics are central to protein function. Due to the uncertainty in selecting protonation states, classical MD simulations are sometimes performed with all amino acids modeled in their standard charged states at pH 7. Here, we performed and analyzed classical MD simulations on high-resolution cryo-EM structures of two large membrane proteins that transfer protons by catalyzing protonation/deprotonation reactions. In simulations performed with titratable amino acids modeled in their standard protonation (charged) states, the structure diverges far from its starting conformation. In comparison, MD simulations performed with predetermined protonation states of amino acid residues reproduce the structural conformation, protein hydration, and protein–water and protein–protein interactions of the structure much better. The results support the notion that it is crucial to perform basic protonation state calculations, especially on structures where protonation changes play an important functional role, prior to the launch of any conventional MD simulations. Furthermore, the combined approach of fast protonation state prediction and MD simulations can provide valuable information about the charge states of amino acids in the cryo-EM sample. Even though accurate prediction of protonation states in proteinaceous environments currently remains a challenge, we introduce an approach of combining p*K*_a_ prediction with cryo-EM density map analysis that helps in improving not only the protonation state predictions but also the atomic modeling of density data.

## Introduction

Within the past decade, the resolution of single-particle cryo-EM structures has improved dramatically, largely due to the improvements in direct electron detectors and processing software.^1^ The resolution of single-particle cryo-EM structures is now comparable to X-ray crystallography and NMR structures, and the so-called “resolution revolution” has made it possible to determine structures of many previously inaccessible complexes, particularly for membrane proteins.^2^ Many of these structures have a resolution better than 2.5 Å, allowing accurate modeling of protein conformations, including ordered water molecules, which has significant implications in drug design^3^ and is also central to our understanding of the molecular mechanism of enzymes that catalyze proton transfer reactions.^4^

Proton transfer can take place across a chain of water molecules via a Grotthuss-type mechanism.^5^ However, proton transfer routes through proteins do not consist only of water molecules but are often made up of hydrogen-bonded networks of polar and charged amino acid residues. Besides titratable residues such as glutamic acid and lysine, which can donate or accept protons, other residues that are part of these networks include asparagine and glutamine, and serine, threonine, and tyrosine with hydroxyl groups in their side chains. These polar residues can not only assist in proton transfer reactions by stabilizing charged intermediates but may also undergo protonation/deprotonation reactions in proteinaceous environments.^4,6−8^ Moreover, titratable acidic and basic residues are well known to act as proton transfer elements and proton loading sites in several enzymes.^4,9,10^ However, the protonation states in protein structures are usually not explicitly modeled. Such information can be obtained with neutron diffraction techniques, but only for relatively small proteins.^11^

The two main structure determination methods for protein complexes, X-ray crystallography and cryo-EM, produce superficially similar results. However, they differ fundamentally in the way the atomic structures are imaged. X-rays are deflected by electrons and thus produce electron density maps, but electrons are scattered by Coulomb potential and are thus sensitive to charges. While positively charged ions just add extra density to an already positive signal, negative charges, in particular O^–^, can give rise to negative electron scattering amplitudes.^12−14^ As a result, negatively charged side chains of glutamate and aspartate residues are not visible in cryo-EM maps. Although the weak density of these side chains has sometimes been interpreted as a result of the radiation sensitivity of the carboxyl group,^15,16^ the former mechanism appears to have a larger contribution.^14,17−19^ The absence of side chain density hampers accurate model building of these negatively charged residues, which are often crucial to protein function. On the other hand, it makes it possible to determine the charge state of acidic residues.

To complement cryo-EM structures, molecular dynamics (MD) simulations are often performed.^20−30^ These help in studying the dynamics of the protein and the solvent as well as the binding and unbinding of lipids, ligands, and ions. The approach of combining structural data with MD simulations is a powerful technique to understand protein structure and function. The abundance of new structures gives plenty of opportunities to perform these simulations routinely at various levels of computational approximations. However, one limitation of conventional molecular dynamics is that covalent bonds are fixed throughout the simulation; therefore, the charge states of titratable residues (often selected as the standard charge state that is Asp/Glu deprotonated, Lys/Arg protonated, and His neutral) remain unchanged. This results in a biased scenario, and biologically relevant conformational states with alternative protonation states are not populated. Moreover, situations where changes in protonation states can occur as a function of conformational dynamics (e.g., membrane proteins catalyzing proton transfer) are also not captured. However, there are various methods that can allow charge states of amino acids to change during MD simulations, such as hybrid QM/MM MD, and constant pH MD, but these are generally computationally costly.^31^

The recent algorithmic developments combined with high computational capacity (including the use of graphical processing units) resulted in constant pH MD simulations to scale as good as conventional MD with only a small computational overhead.^32−34^ However, several challenges remain, as discussed in a recent review by Shen and colleagues,^35^ for instance, adjustment of canonical force field to constant pH simulation conditions to achieve convergence,^36^ which is extremely difficult to achieve in cases when several titratable sites are strongly coupled both electrostatically and dynamically. Another major challenge is to achieve enhanced sampling while also retaining computational speed, especially in cases of sizable protein complexes that undergo large conformational changes. Despite these challenges, constant pH MD simulations have been successfully applied to a variety of small to medium-sized membrane protein systems, including proton/ion channels,^37,38^ antiporters,^39^ and GPCRs,^40^ involving a relatively small number of titratable sites. Nevertheless, for large-scale complexes with hundreds to thousands of titratable sites (such as those studied here), p*K*_a_ prediction with constant pH MD simulations remains a daunting task.

For many proteins, performing conventional MD simulations by systematic altering of charge states of individual titratable residues is unrealistic due to the large number of such residues. Of course, one can alter protonation states of selected conserved residues to study specific questions, and this has indeed been performed to obtain valuable functional insights.^41−45^ However, due to the long-range nature of electrostatic interactions, the protonation state of one titratable residue affects another by ca. 8 kcal/mol (with a separation of 10 Å at ε = 4) and such aspects are often ignored when performing simulations.

Alternatively, one can calculate the p*K*_a_ of all titratable residues in a protein for a given conformational state (obtained from a cryo-EM experiment, for example) and perform MD simulations in that fixed protonation state. There are several methods to perform p*K*_a_ calculations, many of which are based on Poisson–Boltzmann (PB) continuum electrostatics approaches,^46^ for example, the MEAD program.^47^ However, these require a significant amount of preprocessing and can display a large variation in p*K*_a_ values due to subtle conformational changes. Nevertheless, the combined approach of PB-based p*K*_a_ estimation with MD simulations has been used in the past.^48−50^ Alternatively, empirical-based computational methods such as Propka can be employed relatively easily, to give fast and sufficiently accurate predictions of the p*K*_a_ of all ionizable groups present in a protein, even if hundreds in number.^51^ Methods like Propka^52^ often give reasonable estimates for the p*K*_a_ of buried titratable residues, in particular if the sites are far from any redox-active cofactors. Due to their rapid p*K*_a_ estimations, they can be used on thousands of simulation snapshots to obtain profiles of p*K*_a_ change as a function of charge change^53^ and conformational dynamics of protein and ligand.^43,54,55^ Similarly, Monte Carlo-based p*K*_a_ prediction methods have also been used to predict charged states of systems, either using standalone PDB files or simulation snapshots^56^

In this study, we present MD simulations of two high-resolution cryo-EM structures of membrane proteins. The two structures are the respiratory complex I from *Yarrowia lipolytica* (PDB 7O71, EMD-12742)^57^ and the multiple resistance and pH adaptation (Mrp) cation/proton antiporter from *Bacillus pseudofirmus* (PDB 7QRU, EMD-14124).^58^ Both proteins facilitate proton transfer reactions that involve amino acid residues and water molecules, many of which have been resolved in the two structures. The role of protein hydration, water dynamics, as well as change in protonation state is central to the structure and function of these proteins. By performing long-time-scale MD simulations in multiple charge states of these large proteins (total system size of ∼1.3 million and ∼450,000 atoms for complex I and Mrp antiporter, respectively) and extensively analyzing protein and solvent dynamics, we show that both proteins deviate from the original cryo-EM conformation when simulated in the standard protonation state of titratable amino acid residues, while MD simulations in predefined protonation states stabilize the protein conformation much better. We propose that the approach of fast protonation state prediction combined with conventional MD simulations can give insights into the charge state of residues in a cryo-EM structure. Furthermore, we find that the prediction of protonation states of acidic residues agrees well with the charge state assignment based on cryo-EM density maps and that outliers can be identified with the approach discussed here, leading to an improved modeling of cryo-EM density data.

## Methods

We performed all-atom molecular dynamics simulations of complex I from *Yarrowia lipolytica* (PDB: 7O71)^57^ and Mrp antiporter from *Bacillus pseudofirmus* (PDB: 7QRU, altloc B).^58^ Both proteins were placed in a mixed lipid bilayer and solvated with TIP3P and NaCl. Gromacs versions 2020 and 2021^59^ were used for minimization, equilibration, and production runs. Full details of the molecular dynamics setups can be found in previous work on complex I^57^ and Mrp antiporter.^58^ The production runs were performed without any constraints in an NPT ensemble, using Nosé–Hoover thermostat^60,61^ at 310 K and Parrinello–Rahman barostat^62^ at 1 atm. We employed the LINCS algorithm to achieve a 2 fs time step,^63^ and the particle-mesh Ewald method^64^ with a cutoff of 12 Å to handle electrostatic interactions. The cutoff for van der Waals interactions was 12 Å, with a switching distance of 10 Å. Table S1 lists the simulation setups, and the S and P7 states for complex I and Mrp antiporter are extended simulations from previous work. The P5, P6, P8, and P9 simulations of Mrp are new systems constructed using the same protocol.

Estimates of p*K*_a_ were performed using the Propka software package.^51^ The calculations were performed on the PDB structures of complexe I and Mrp antiporter. Asp, Glu, and His were considered protonated (neutral) if their p*K*_a_ was more than 7 in the P7 simulations, while Lys was considered deprotonated if its p*K*_a_ was less than 7. The same process was performed in the P5, P6, P8, and P9 setups with their respective p*K*_a_ cutoffs changed. His was considered with the δ-nitrogen protonated when neutral. p*K*_a_ calculations were performed on the original high-resolution structural data, and not on the MD simulation snapshots, even though the latter can be informative of p*K*_a_ changes as a function of charge and conformational change.^43,53−56^

Exponential fit of hydrogen bond decay was calculated using the SciPy *curve_fit* function.^65^ We used a monoexponential of the form *y* = *me*^(−*tx*)^ + *b*, where *m*, *t*, and *b* are parameters that could vary. The quality of the fit was determined using *R*^2^, obtaining values >0.9. The half-life was calculated as *t*_1/2_ = *t* × ln 2.

The statistical significance of the results was checked using the distributions shown in Figure 2. This was evaluated with both the *t* test and Kolmogorov–Smirnov-test, and for both the protein systems, complex I and Mrp antiporter, low *p*-values (<0.001) were obtained. Additionally, we tested the *p*-values using a smaller set of random data from the distributions (500 out of 3000 data points), and the *p*-values still remained low (<0.001).

The cryo-EM density analysis and remodeling of the resolved structures were done using Coot.^66^ The change in the glutamic and aspartic acid conformations was achieved with the rotamer function in Coot. The key requirement for the new position of the side chain was a substantial deviation from its previous position, as well as avoiding clashes with its surroundings. Care was taken not to position the side chain in positive density.

Visualization, analysis, and figure preparation were performed using VMD,^67^ Pymol,^68^ and UCSF Chimera.^69^

## Results

**Figure 1:**
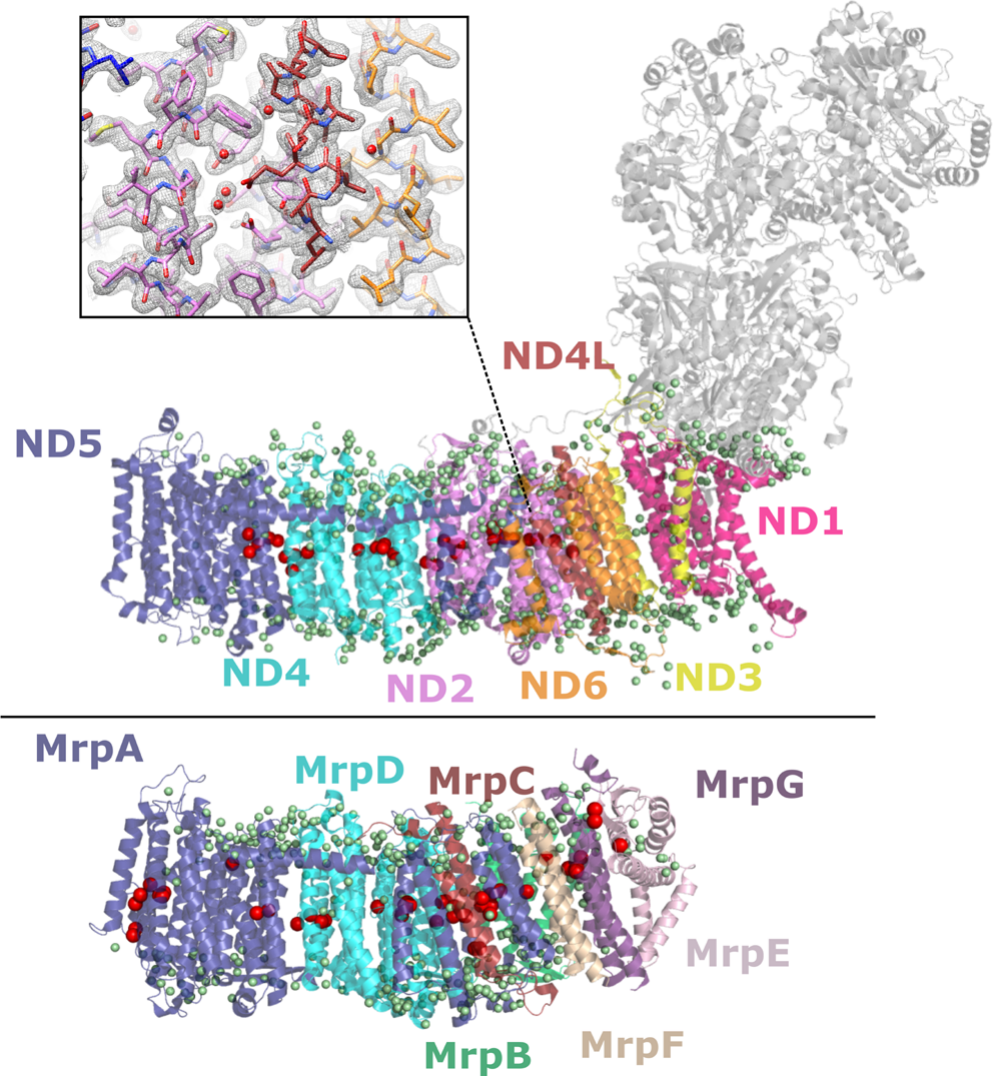
Structure of the membrane domain of respiratory complex I (top) and Mrp antiporter (lower). The protein is shown in ribbon representation and colored by subunit. Only core membrane-bound subunits are shown in color for complex I, with the hydrophilic core subunits shown as gray. Structurally resolved water molecules are shown as small green spheres, with those in the functionally relevant hydrophilic axes of complex I and Mrp antiporter shown as larger red spheres. Proton translocation is suggested to take place across this axis, which is a vital part of the mechanism for both proteins. The inset in the upper panel shows a region of the complex I membrane arm within the high-resolution cryo-EM density (gray mesh), including density for water molecules.

Two high-resolution membrane protein structures were chosen for this investigation. First, the 2.1 Å resolution structure of complex I from *Y. lipolytica*, which is almost 1 MDa in size, and has more than 1600 structural water molecules resolved.^57^ Around 100 of these are in the potential proton transfer pathways present in their membrane-bound subunits (Figure 1). Second, a structure from the evolutionarily related Na^+^/H^+^ Mrp antiporter^58^ at a similar resolution of 2.2 Å. This protein is much smaller than complex I (ca. 213 kDa) and consists of membrane-bound subunits only, without redox-active cofactors. There are 360 water molecules resolved in the structure of the Mrp antiporter, around 70 of them in the potential proton and sodium transfer pathways (Figure 1). The structure of complex I contains more than 1300 titratable residues, while the Mrp structure has around 200. We performed MD simulations on both complexes with all titratable sites either modeled in their standard states (S state) or based on p*K*_a_ calculations at pH 7 (P7 state). We find that in the P7 state, complex I has 68 neutralized titratable amino acids, out of which 25 are in the membrane core of the enzyme (Figure 1), while in the Mrp antiporter 13 residues are neutralized in the P7 state. Accordingly, the charge reduces from +90/–94 to +83/–76 in the complex I membrane arm and from +116/–118 to +110/–112 in the Mrp upon neutralization of titratable sites as part of p*K*_a_ calculations (see Tables S2 and S3 for lists of residues neutralized in complex I and Mrp antiporter, respectively).

### Global and Local Mobility of Proteins in P7 and S States

**Figure 2:**
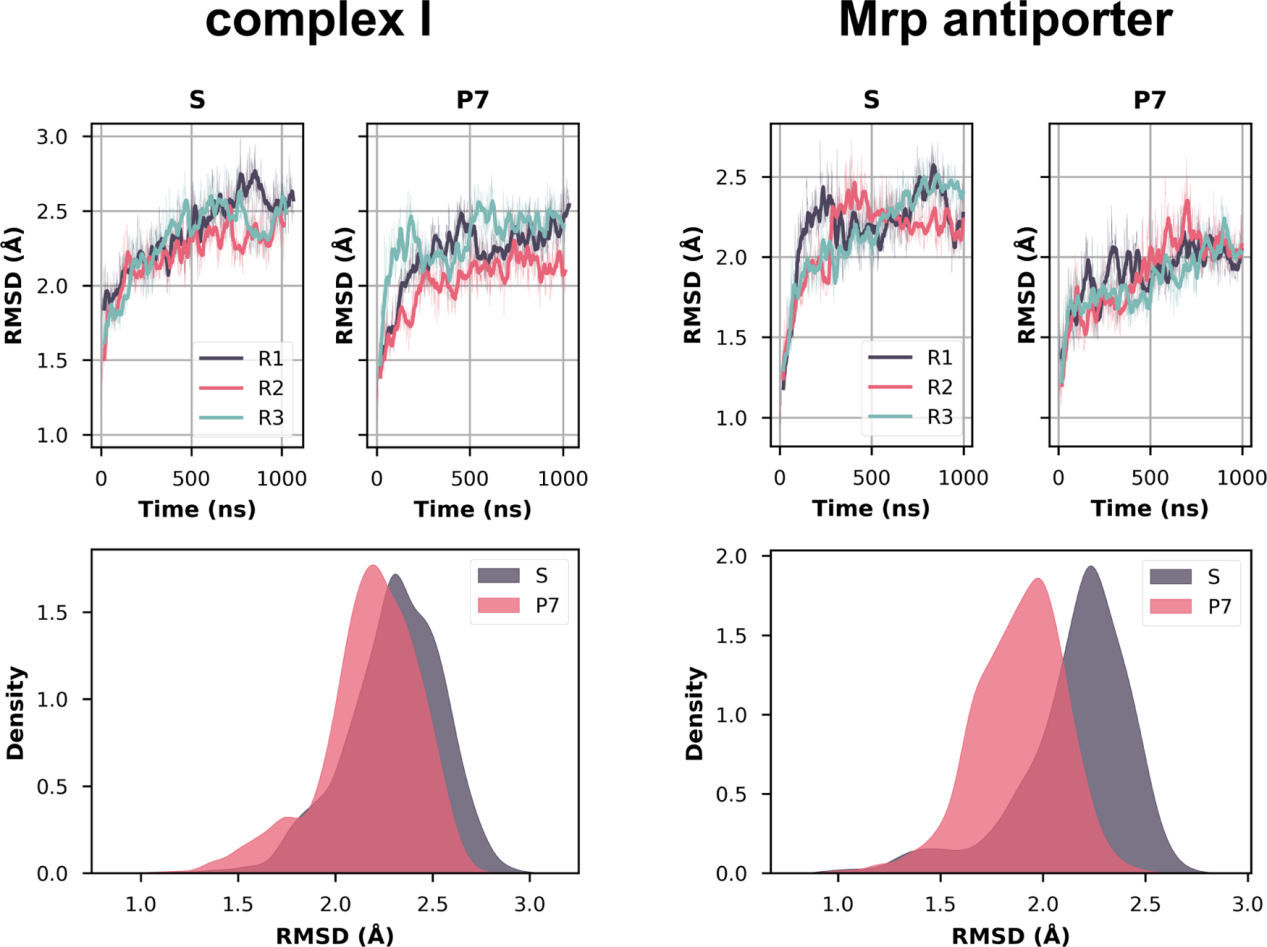
RMSD (root-mean-square deviation) of the backbone atoms over time for both complex I (core membrane subunits, left) and Mrp antiporter (right) in the S and P7 states. The upper plots show the RMSD as a time series, with each colored trace representing a different simulation replica. The thick lines show the moving average of 20 ns, while the thinner lines show the RMSD for every 1 ns. The bottom panels show the distribution of RMSD values in the S and P7 states using a kernel density estimate (KDE) function with combined data of all three replicas (see the Methods section for additional statistical analysis).

First, the overall global mobility of both systems was analyzed in the S and P7 states. Figure 2 shows the time series as well as the distribution of the RMSD (root-mean-square deviation) of the backbone atoms for the core membrane-bound subunits of complex I and Mrp antiporter in the two simulation states. The P7 state systems with lower charge have overall lower RMSD for both protein complexes (Figure 2, see also the Methods section), which means that the backbone atoms stay closer to the starting conformation throughout the simulation. This notion is also true if a similar RMSD analysis is performed on the Cα backbone atoms and all protein atoms, excluding hydrogens, even with the inclusion of the hydrophilic domain of complex I (Figure S1).

**Figure 3:**
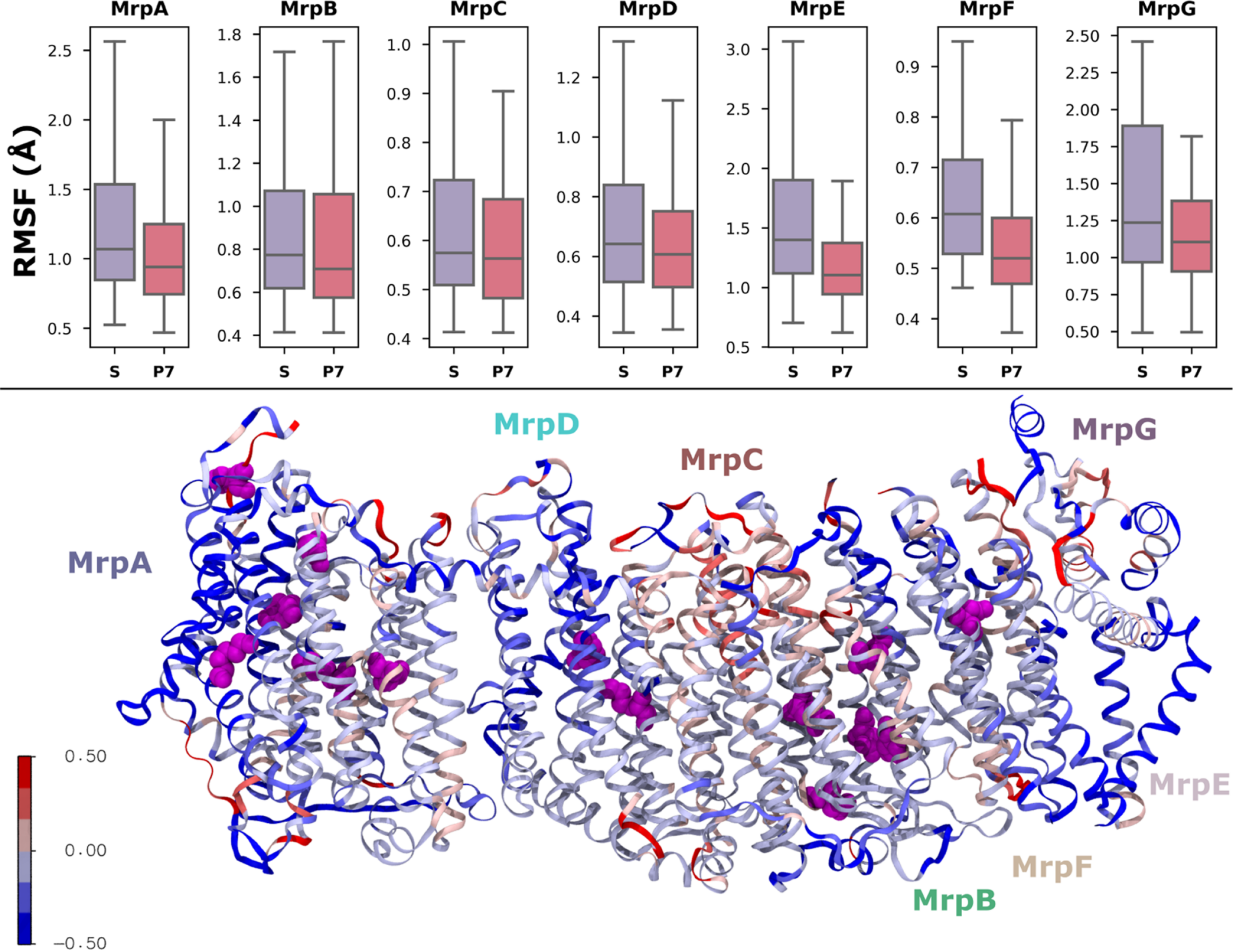
(Top) RMSF (root-mean-square fluctuation) of Cα atoms in Mrp antiporter subunits, calculated for all simulation data in the S and P7 states. The box plots represent the distribution of RMSF values for each subunit, in both states. The shaded box represents the interquartile range, with the middle line showing the median. The upper and lower lines are the maximum and minimum values, respectively. (Bottom) Protein structure colored according to the extent of conformational change in the P7 state compared to the S state. Red represents Cα RMSF increased in the P7 state, and blue represents a decrease. The magenta spheres show the positions of residues that undergo a protonation state change in P7 simulations.

Similarly, the RMSF (root-mean-square fluctuation), which measures the average amount that an atom moves during the entire simulation, is also consistently found to be lower in the P7 states than in the S states. The box plots in Figure 3 show the RMSF of Cα atoms in each individual subunit of the Mrp antiporter. For all antiporter chains, lower RMSF values are observed in the P7 state, indicating that there is overall stability in the system as charges of titratable sites are neutralized (based on p*K*_a_ estimates). A similar trend was also observed in complex I (Figure S2). The spatial distribution of the RMSF data is shown in Figure 3 (bottom). MrpA subunit (Figures 1 and 3) has the largest number of titratable residues that undergo protonation state changes to become neutralized, and this has a clear impact in reducing the RMSF of the subunit. Interestingly, the stability of the protein also increases in areas somewhat far from the residues that change protonation states (magenta spheres), highlighting that the charge change of just a few titratable residues can have conformational changes imparted both at the local and the global level.

In addition to the changes at the backbone level, the side chain conformations of individual residues were also analyzed (Figure S3). We find that the side chains of several titratable residues in both proteins remained closer to the structural conformation in P7 states in comparison to S states, as shown by RMSD and side chain rotamer analysis (Figure S3). Overall, the key observation is that MD simulations performed in predefined protonation states based on fast proton affinity calculations retain a structure closer to the cryo-EM conformation for both protein complexes studied here. This in turn means that the simulated charge state is closer to the charge state of the protein during the cryo-EM sample preparation. To understand why P7 state simulations show overall stability relative to that of the S state, we performed additional analyses.

### Water–Protein Interactions and Protein Hydration in P7 and S States

**Figure 4:**
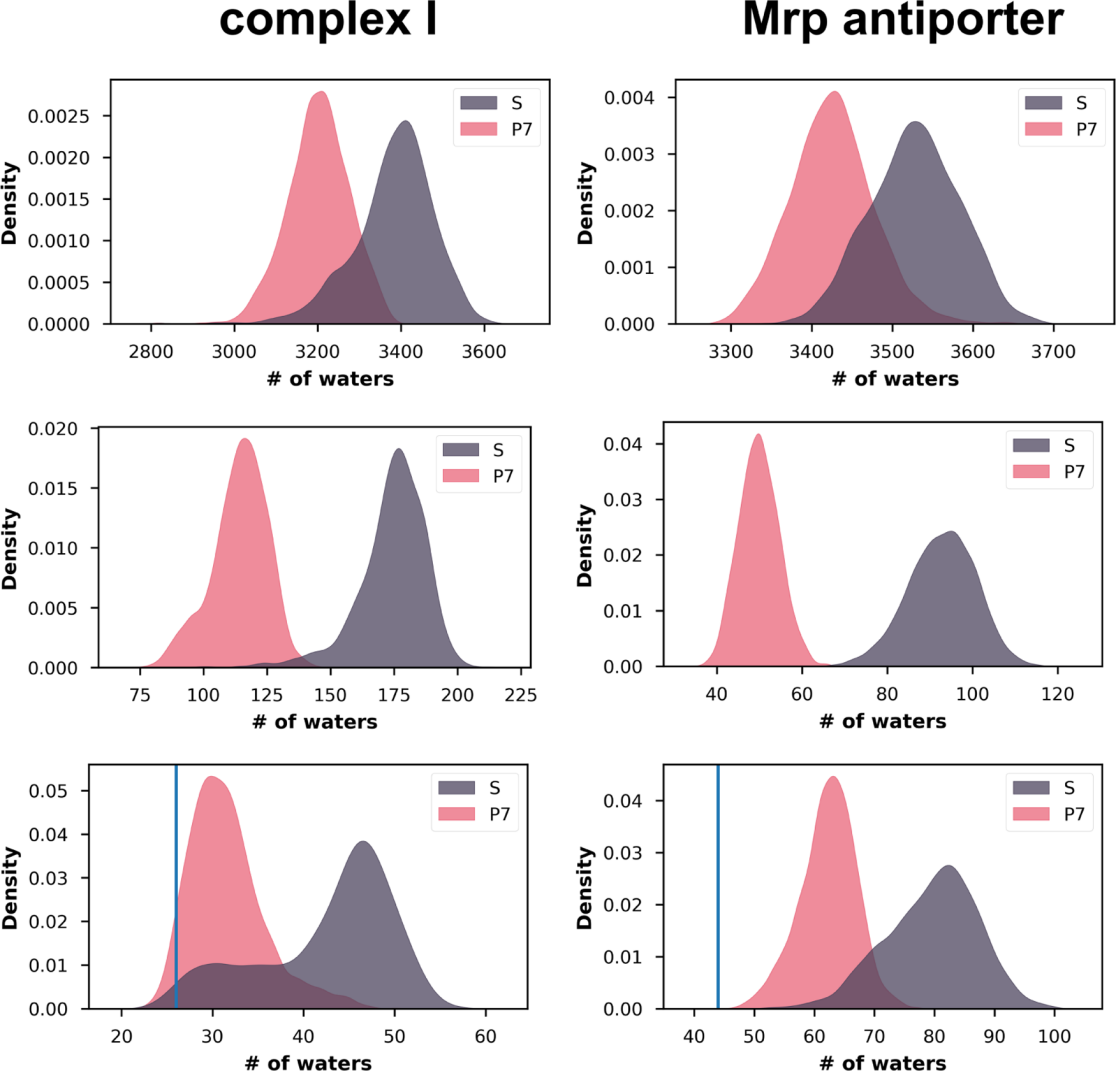
KDE plots for the number of water oxygen atoms in contact within 4 Å of protein residues, with the left column showing data for complex I (core membrane subunits) and the right column showing data for the Mrp antiporter. Top panels show the number of waters in contact with all protein atoms, middle panels show protein residues that change protonation state in P7 state simulations, and bottom panels show residues in the functionally relevant horizontal axis (Figure 1). Blue vertical lines in the lower panels indicate the number of waters resolved in the cryo-EM structures that are in contact with the residues in the central hydrophilic axis.

In the high-resolution structures of complex I and the Mrp antiporter, the positions of several water molecules are resolved. We therefore next analyzed how water–protein interactions and protein hydration are affected in the S and P7 simulation states. The contacts between all water molecules and protein were clearly reduced in the P7 states compared with the S states (Figure 4, top). The lower charge in P7 states (+83/–76 compared to +90/–94 in the S state of complex I and +110/–112 compared to +116/–118 in the S state of Mrp antiporter) prevents extensive hydration of the protein, resulting in a lower number of contacts between water oxygens and protein. This is also reflected in the clear reduction of water contacts that take place with the residues that change protonation states (Figure 4, middle).

In both proteins, a hydrophilic axis runs through the central part of the membrane domain, which is thought to be the structural basis for proton transfer reactions.^57,58,70−73^ We next evaluated the time evolution of internal water molecules in this hydrophilic axis for both states of both proteins. We observed that the number of internal water molecules in the P7 state remains lower and closer to the water content observed in the structures, whereas in S state runs, extensive hydration of the proteins is observed (Figure 4 (bottom) and Figure S4).

**Figure 5:**
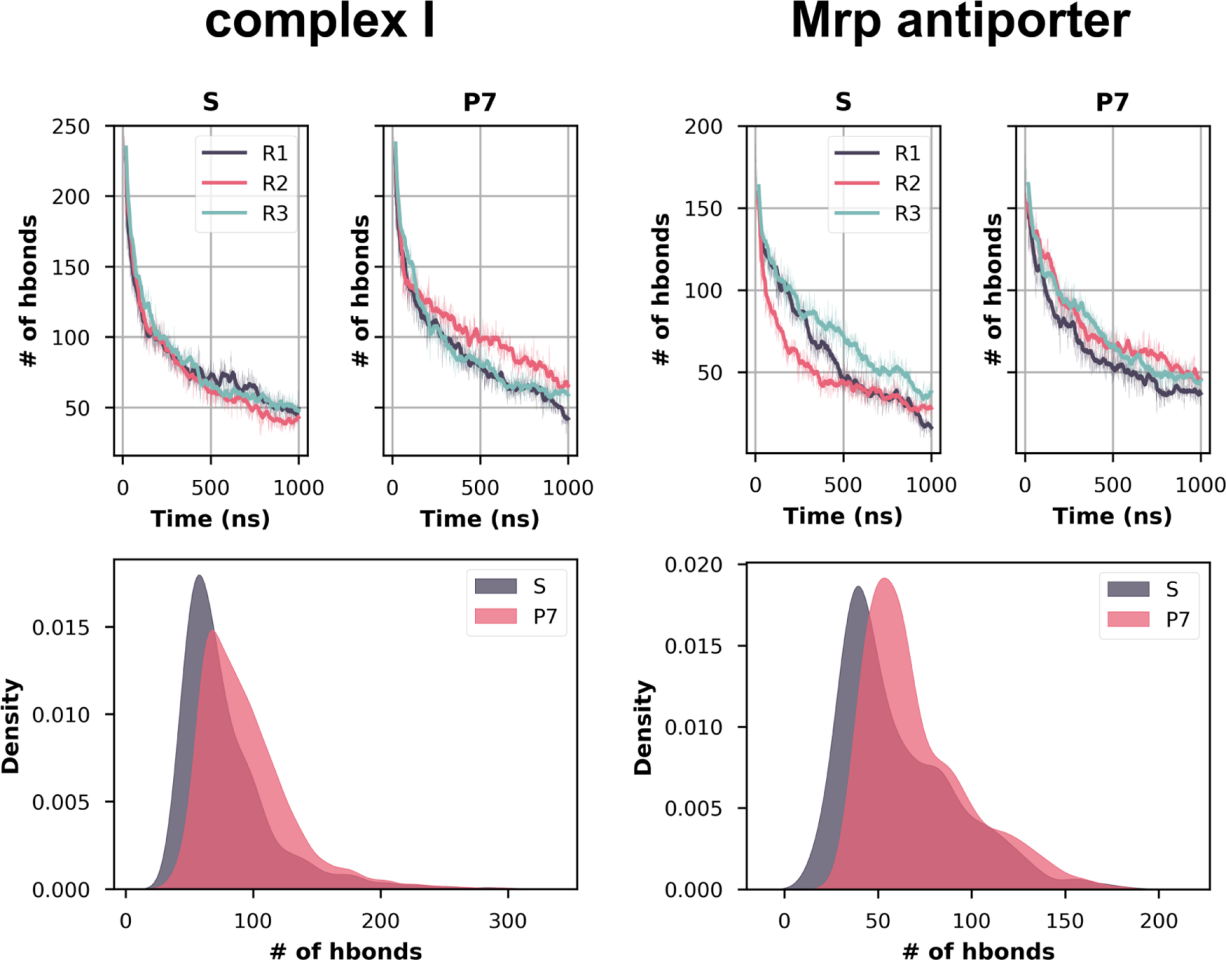
Hydrogen bonds between protein and structural water molecules. (Top) The number of hydrogen bonds throughout the S and P7 simulation trajectories is shown as a time series. The different colors of traces represent different simulation replicas. (Bottom) The data from all trajectories shown as density. Only the core membrane subunits of complex I were considered for analysis. The hydrogen bonding distance was cut off at 3.5 Å and the angle at 150°.

Next, we analyzed the hydrogen bonding between the water molecules and the protein. The time evolution of the hydrogen bonds between protein and structurally resolved water molecules showed a more rapid decline in the S states compared to the P7 states, with the latter also displaying a slightly higher number of such interactions on long time scales (Figure 5). This indicates that although water exchange does take place in the P7 state, the exchange is less rapid and a greater number of hydrogen bonds that existed in the initial structural state are maintained for long time scales. The stable water–protein hydrogen bonds combined with lower hydration in the P7 state also suggest a lower water exchange rate in these simulations. The details of which protein-structural water hydrogen bonds are stabilized in the Mrp antiporter are shown in Table S4, and notably, the stabilization of these hydrogen bonds is associated with a decrease in RMSF.

In Mrp antiporter, in the S state, most structural waters lose hydrogen bonds with the protein residues in the μs time scale, whereas in the P7 state, the loss is relatively slower and less extensive in the given time scales (Figure 5). Interestingly, the loss of hydrogen bonds is much more drastic in complex I simulations, especially in the shorter time scales. To further probe into the kinetics of loss of hydrogen bonding between the structural waters and protein, we fitted the profiles (Figure 5, top) to exponential function (see the Methods section) and obtained the half-life of protein–water hydrogen bonds (*t*_1/2_). The results show that on average in complex I P7 state hydrogen bonds survive longer (average *t*_1/2_ ∼ 162 ns) compared to the S state (average *t*_1/2_ ∼ 113 ns). For relatively shorter time scales (ca. 500 ns), a more subtle but similar effect was also observed for Mrp antiporter simulations (average *t*_1/2_ ∼ 135 ns in the S state compared to ∼145 ns in the P7 state).

### Protein–Protein Interactions

**Figure 6:**
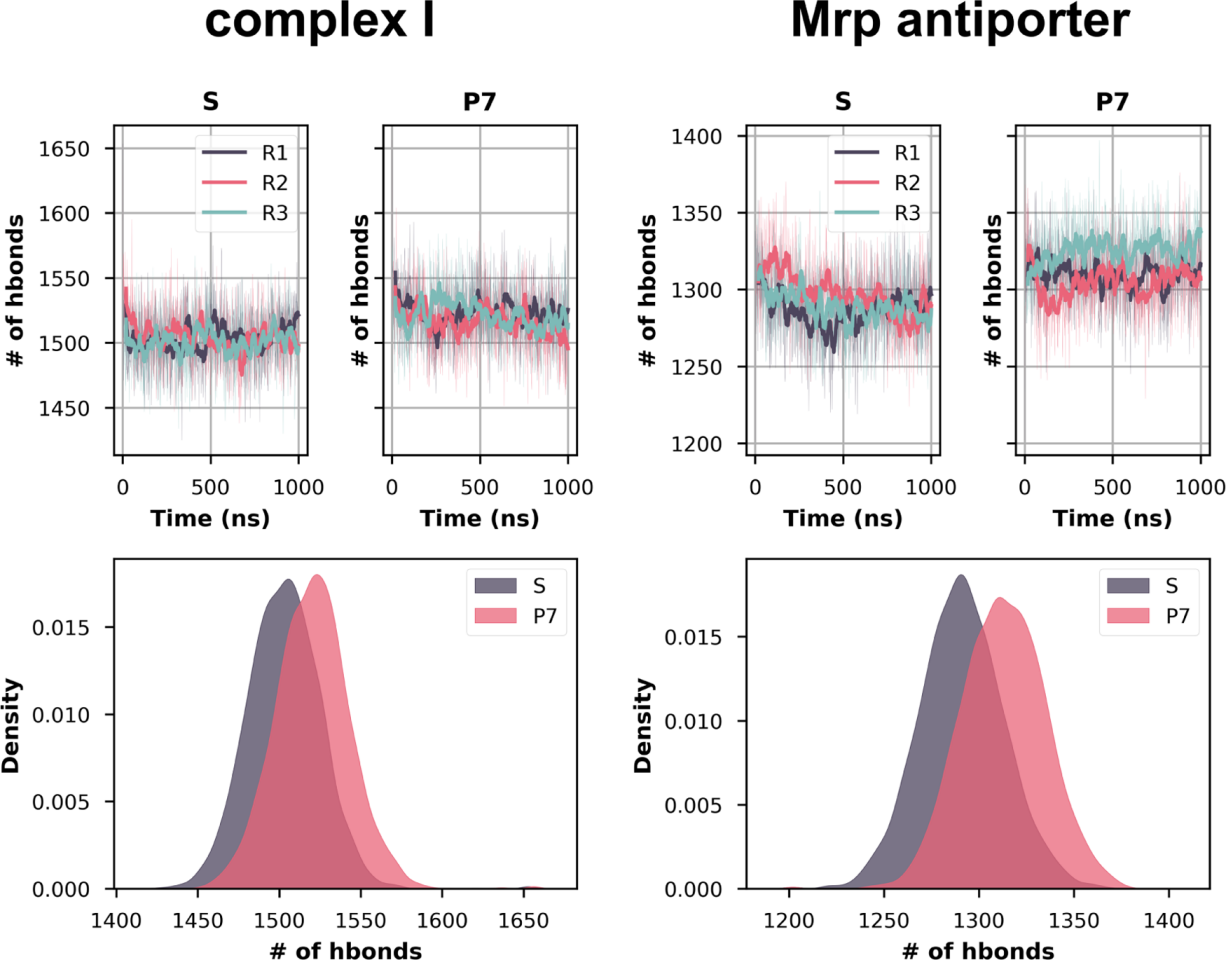
Number of hydrogen bonds between protein side chains throughout the simulation for complex I and Mrp antiporter. Top panels show the time evolution of all hydrogen bonds for three different simulation replicas, with the hydrogen bonding distance cut off at 3.5 Å and the angle at 150°. Bottom panels show the same data as KDE plots. In the case of complex I, only core subunits of the membrane arm are considered (see Figure S5 for full protein).

In addition to the protein–water hydrogen bonding interactions, protein–protein hydrogen bonds were also analyzed. The number of protein–protein side chain hydrogen bonds is consistently found to be higher throughout the P7 state trajectories (Figure 6). This implies that structural stability is higher in P7 states compared to the S states for both Mrp antiporter and complex I, as also observed in RMSD and RMSF analysis (see above). The lower number of protein–protein interactions is also in agreement with the higher level of hydration observed in the S state. In other words, enhanced water entry/exit in the protein causes protein–protein interactions to be perturbed, resulting in structural destabilization. Specific pairs of residues from the Mrp antiporter that showed a significant increase (>50%) in hydrogen bonding are shown in Table S5. Interestingly, although only two of the residue pairs undergo changes in the protonation state between S and P7 states, still several other pairs show enhancement in hydrogen bonding occupancy, pointing out that long-range effects can occur upon charge change.

The above data on protein dynamics and hydration, as well as hydrogen bonding analysis in two different charge states, lead to the conclusion that higher protein fluctuations are caused by extensive hydration, which in turn is caused by the highly charged state of the protein, as in the S state. The correct (or near-to-correct) charge state of the protein is thus critical for its structural stability. Hence, prior to launching long-time-scale MD simulations of high-resolution membrane proteins, a proper charge analysis by means of p*K*_a_ calculations is recommended.

### MD Simulations with Protonation States Set at Different pH Values

**Figure 7:**
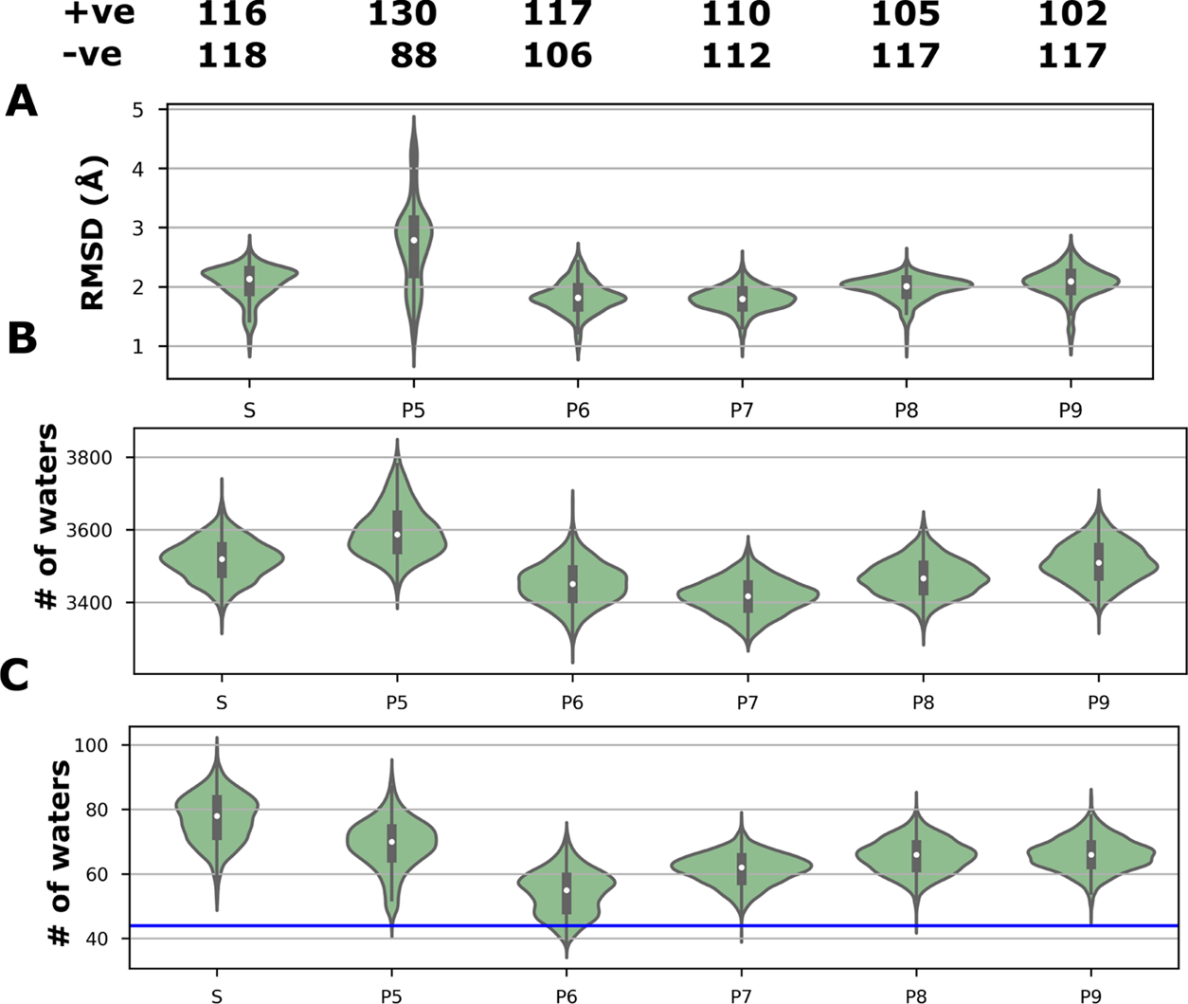
Comparison of Mrp antiporter MD simulations with fixed protonation states set at different pH values (based on p*K*_a_ calculations). (A) RMSD of backbone atoms, (B) number of water molecules within 4 Å of all protein residues, and (C) number of waters in the functionally relevant central axis (Figure 1), with the blue line showing the number of waters in contact with protein observed in the cryo-EM structure. Data are shown as violin plots, where the shaded area is a KDE plot of all trajectory data and in the center is a box plot displaying the median (white dot with the black box indicating the interquartile range). The total number of positively and negatively charged residues in the different simulation states is listed above (A).

It is clear from the above analysis that the protein simulations that have charge states defined by predetermined protonation states of titratable residues set at pH 7 are well behaved and are closer to the conformation obtained from cryo-EM. This is true for both global and local mobility as well as protein hydration. To further explore how altering the protonation state affects these characteristics, we performed a series of simulations of the Mrp antiporter complex with the protonation states based on four more pH values: 5, 6, 8, and 9. Note that these are still conventional classical MD simulations where the protonation states of the residues are fixed. The simulations indicate that the RMSD of the protein (Figure 7A) was lowest at pH 6 and pH 7, with pH 5 showing the most instability. Similarly, both the number of water molecules in contact with protein (Figure 7B) and the number of water molecules in the central hydrophilic axis (Figure 7C) were lowest for pH 6 and 7, with pH 5 deviating the most. At pH 5, many of the negatively charged residues become neutral, and positive residues stay positive. Importantly, many histidine residues become doubly protonated, which introduces excess positive charge in several regions of the protein. As a result, bulk water molecules enter the protein, causing instability in protein side chains and loops, even more than in the S state.

### Charge State Assignment Based on Cryo-EM Density

The above analysis highlights the importance of the correct charge state description of the protein, which is necessary for its stability during MD simulations. However, the prediction of the charge state by p*K*_a_ calculations has its limitations. We thus attempted to deduce the protonation states of amino acids from the high-resolution cryo-EM data. Cryo-EM density maps can be used to identify the charge states of acidic amino acid residues (glutamates and aspartates) because neutral and negatively charged atoms have vastly different scattering amplitudes^14,17^ and thus different cryo-EM density profiles. By carefully analyzing the cryo-EM density in our high-resolution maps, we obtained the charge states of selected glutamate and aspartate residues and compared those to the p*K*_a_ values obtained based on the modeled structure. We found that there was an agreement between the charge state assignment based on the cryo-EM maps (EMD-12742 for complex I and EMD-14124 for the Mrp antiporter) and the predicted p*K*_a_ based on the derived atomic models (PDBs 7O71 and 7QRU, respectively). However, several acidic residues showed discrepancies; the p*K*_a_ calculations predicted some carboxylates to be charge neutral that were assigned to be anionic by cryo-EM density analysis (Table 1). A possible explanation for this discrepancy is that although the protein model-based p*K*_a_ predictions are largely correct (especially for buried residues), the positions of the (invisible) side chains of charged glutamates and aspartates were incorrectly modeled, resulting in overestimated structure-based p*K*_a_s. To fix this discrepancy between the two estimates, the cryo-EM density map of respiratory complex I (EMD-12742) was revisited. We remodeled the side chains of selected residues that are predicted to be anionic based on missing cryo-EM densities using Coot^66^ and re-performed the p*K*_a_ prediction on the new model. A total of 7 glutamates and aspartates were remodeled one at a time (Table 1).

**Table 1:** Charge State of Selected Acidic Amino Acid Residues in Complex I from Cryo-EM Density Data (EMD-12742) and p*K*_a_ Calculations, Based on PDB 7O71 Coordinates or on a Remodeled Carboxylate Side Chain^a^

	density charge estimate	original p*K*_a_	remodeled p*K*_a_
E147^ND1^	charged	9.11	7.03
E206^ND1^	charged	8.85	6.37
E210^ND1^	charged	7.96	5.69
E231^ND1^	charged	7.44	6.56
D67^ND3^	neutral	7.18	9.34^b^
E69^ND3^	charged	9.27	9.16
E30^ND4L^	charged	7.59	8.69
E66^ND4L^	charged	9.81	10.16

a All carboxylates were remodeled independently.

b D67^ND3^ was not remodeled, but instead the new p*K*_a_ value is based on the structure with E147^ND1^ remodeled. Note the calculated p*K*_a_ values are instantaneous proton affinities corresponding to the original or remodeled conformation.

**Figure 8:**
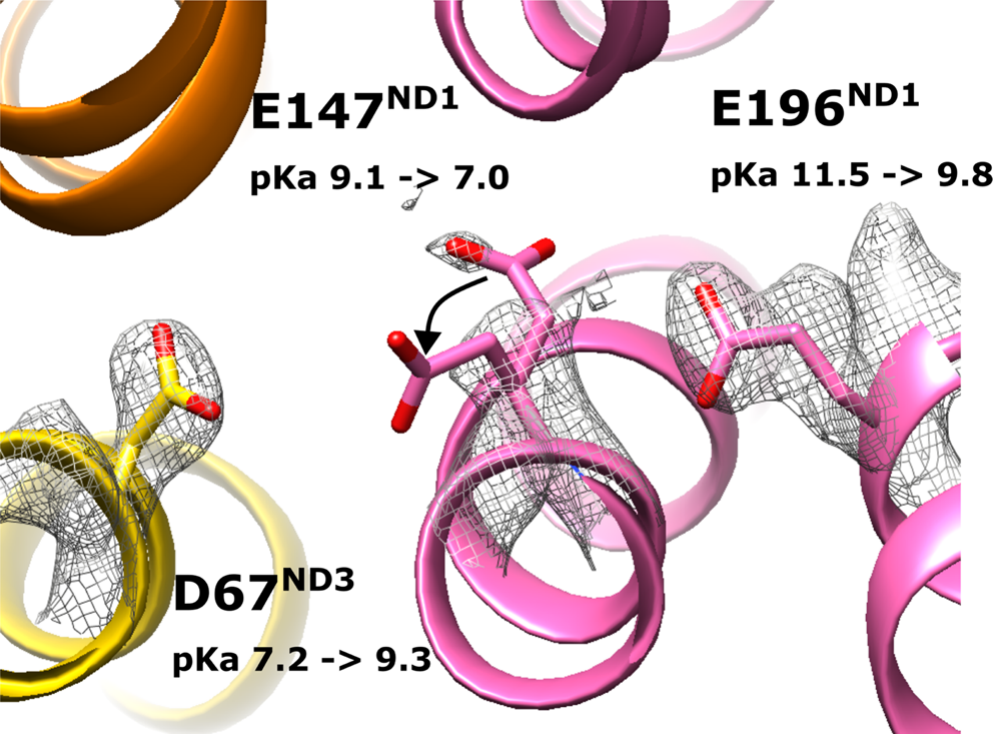
Remodeling of the side chain of E147^ND1^ (indicated by black arrow) improved its p*K*_a_ as well as that of neighboring D67^ND3^. The first value of p*K*_a_ is based on the original coordinates (PDB 7O71), while the second value is after remodeling the side chain of E147^ND1^. The cryo-EM density near the three residues is shown as gray mesh. Note that in the cryo-EM structure, the negatively charged side chain of E147^ND1^ was modeled in positive (noise) density.

The residues E231^ND1^ and E147^ND1^ of the functionally relevant E-channel in complex I^57^ were both predicted to be neutral based on p*K*_a_ calculations but charged based on density analysis. When p*K*_a_ calculations were performed on remodeled side chains, it led to a drop in their side chain p*K*_a_ and stabilization of their deprotonated states in good agreement with cryo-EM density-based assignment (Table 1). Interestingly, the remodeled side chain conformation of E147^ND1^ not only improved the p*K*_a_ of the residue itself but also that of residues in its surroundings, e.g., D67^ND3^ (Figure 8, Table 1). A similar improved agreement between p*K*_a_ prediction and cryo-EM-based charge state assignment was also observed for E206 and E210 from the ND1 subunit (Table 1), both of which are known to be central for protein function.^57^ However, p*K*_a_ predictions of E30 and E66 from ND4L, as well as E69 from ND3 could not be improved despite their remodeled side chains. One potential reason for this could be that the surroundings in two different conformations do not differ as drastically as those in the other cases. Furthermore, the approximate nature of p*K*_a_ predictions is well known and with no explicit treatment of neighboring solvation can result in the overestimation of p*K*_a_ values. All in all, remodeling of several side chains not only improved the charged state assignment but also improved the atomic modeling of cryo-EM density data (see the Discussion section).

## Discussion

Protonation states of amino acids play a key role in protein structure stability and function. However, it is extremely challenging to obtain an accurate estimate of the p*K*_a_ of an amino acid both by experiments as well as by computations, and as a result in most cases, p*K*_a_ values and how they change during enzymatic action remain unknown. Because of this, MD simulations are often performed by modeling amino acids in their fixed protonation states, including standard charged states. Here we show that MD simulations of a membrane protein with all amino acids modeled in their standard charged states result in lower stability of the protein overall and drive the conformation of the protein far from its starting point, the cryo-EM conformation. Instead, if an MD simulation is performed by fixing the charged states of titratable amino acids based on p*K*_a_ calculations, the conformational state remains closer to the original structure. We note that previous work has also highlighted the importance of p*K*_a_ estimates prior to MD simulations in different combinations of protonation states, especially when it comes to the functionally relevant regions of the protein. These studies helped in elucidating the importance of amino acid protonation states on ligand binding,^74^ drug transport,^75^ ion binding,^76^ reaction mechanism,^77^ and functionally relevant conformational dynamics.^78,79^ In this study, we have focused on two very large bioenergetic proteins for which high-resolution structures are available and show the importance of correct protonation state assignment on conformational dynamics of these large protein complexes.

Our analysis of protein mobility and hydration as well as residue–water and residue–residue interactions supports the notion that protein conformation diverges more when all titratable sites are modeled in their standard states. Also, since the structural conformation is retained in MD simulations performed after p*K*_a_ calculations, we propose these more closely match the charge states of amino acids that existed during the cryo-EM experiment. While similar methods of approximate proton affinity calculations combined with MD simulations have previously been used^48−50^ (see also above), we note that this approach is especially important when dealing with such large proteins (>1000 titratable residues) with substantial membrane domains. Our results also indicate that at least in the systems studied here the relationship between protein stability and hydration is strong and that reduced hydration leads to a more stable simulation system which can maintain important interactions. When leaving all protonatable residues in their standard charged states, hydration is considerably enhanced, resulting in important protein–protein interactions being broken, thereby reducing stability. Even though we have focused on bioenergetic proteins in this study, similar observations may hold true for other large membrane protein systems.

The higher level of hydration observed in the standard state simulations is primarily due to the charged states of buried titratable amino acid residues. These charged amino acid residues in the low protein dielectric interior create an unfavorable high-energy scenario due to poor solvation. This establishes an electric field, pulling bulk water molecules into the protein and enhancing solvation similar to the nanoscopic electroporation described earlier,^80^ see also ref (41). We also note that protein hydration obtained from MD simulations performed in predefined charge state is much closer to the structural hydration than that from the MD simulations performed in standard state of the residues. Despite this, the hydration obtained from MD simulations is, in general, higher than the hydration observed in the structure. The reason for this discrepancy is in part due to the underestimation of protein hydration in the cryo-EM maps, where only highly occupied and tightly bound water molecules can be observed.

By performing MD simulations in predetermined charge state from p*K*_a_ calculations, one not only investigates the state that was captured during structure resolution but also its time evolution more accurately. This is particularly useful in situations where there are a particularly large number of titratable residues present in the protein with possible functional relevance, such as in photosynthetic and respiratory enzymes as well as proteins that couple translocation of ions or metabolites to proton transfer reactions. However, empirical p*K*_a_ calculations or more thorough continuum electrostatics-based p*K*_a_ estimations have limited accuracy, and site–site interaction energy terms can often dominate, leading to large-scale p*K*_a_ shifts with subtle changes in structure. Thus, it is important to use an accurate input model, based on high-resolution data where there is less uncertainty in side chain placements. Below ∼2.5 Å resolution the correct rotamers of most side chains can be confidently modeled, with the key exception being negatively charged side chains in cryo-EM maps, which have negative atomic scattering factors^13,17,18^ and thus are usually invisible beyond the Cβ atoms. As modeling and refinement programs do not take this into account, the side chain orientation of these residues is unreliable,^18^ thus leading to questionable results from p*K*_a_ predictions and MD simulations. On the other hand, this fact makes it possible to deduce the charge state of acidic residues from the cryo-EM density.

To validate the protonation state predictions with experiments is a challenging task, especially for massive enzymes like respiratory complex I, which has many electrostatically coupled titratable sites that are also located in functionally critical regions of the protein. However, in some specific cases, experimental-computational agreement of protonation state prediction has been achieved, for example by comparing calculated C=O stretching frequencies to those from Raman spectroscopy.^48^ Additionally, site-directed mutagenesis studies combined with spectroscopic measurements have also shed light on the p*K*_a_ of buried titratable residues, which have also been evaluated computationally, reaching agreement.^81,82^

We note that an exhaustive sampling of the charge and conformational states of all titratable sites is necessary to obtain their correct protonation states for a given protein structure. However, this is nontrivial, especially in cases when there are a large number of titratable sites and when several titratable sites are coupled strongly both electrostatically and dynamically, which is the case in the proteins studied here. Empirical protonation state prediction methods such as Propka^51^ are fast approximations to obtain proton affinities and can be applied on protein structures and snapshots from MD simulations to obtain mechanistic insights.^44−55^ However, in difficult cases such as of coupled titratable sites,^83^ their accuracy can be limited. The more accurate constant pH MD simulations strive to achieve this,^84^ and combined with rapid methodological advancements,^32,34^ such techniques have indeed been applied to various relatively small to medium-sized membrane protein systems,^37−40,85^ but several challenges remain.^35,36^ We envisage that in such difficult cases, the alternative of fixing protonation states of amino acid residues is a reasonable way forward, which can even be used together with constant pH approaches. All in all, a sound protonation state prediction is required that reflects the high occupancy (low energy) conformations of amino acids observed in the structure, which can be achieved by a combination of experimental and computational methods, as discussed here.

Here, we propose a method that may assist to improve modeling of side chains of acidic residues. By performing fast empirical p*K*_a_ calculations on the 3D model, pKas of all acidic residues can be estimated. If the predicted p*K*_a_ value suggests a neutral state of an acidic residue that is expected to be anionic based on density analysis, this warrants remodeling of the side chain of the amino acid. If the remodeled side chain conformation yields a lower p*K*_a_, this is likely to be the more accurate conformation. By combining p*K*_a_ calculations with side chain remodeling on selected acidic residues of complex I, we show here that this is feasible. This is a vital step in improving the reliability of cryo-EM models for negatively charged side chains. In our future work, we aim to automate this process and obtain more accurate models of side chains of acidic amino acid residues from the cryo-EM data, thus creating a feedback loop between structural biology and computer simulations.

## Supporting Information Available

RMSD for complex I and Mrp antiporter systems (Figure S1); RMSF for complex I (Figure S2); side chain dihedral and RMSD (Figure S3); central axis hydration (Figure S4); hydrogen bond evolution for complex I (Figure S5); list of simulation setups (Table S1); protonation states of titratable residues in MD simulations for complex I (Table S2) and Mrp antiporter (Table S3); and hydrogen bond occupancy in Mrp antiporter (Tables S4 and S5) (PDF)
