# Supplementary material for: Role of Protonation States in the Stability of Molecular Dynamics Simulations of High-Resolution Membrane Protein Structures
Source: J Phys Chem B. 2024 Mar 2;128(10):2304–16. doi: 10.1021/acs.jpcb.3c07421 (PMC11389979; doi:10.1021/acs.jpcb.3c07421)
Supplement: Supplementary file 1 — jp3c07421_si_001.pdf [file jp3c07421_si_001.pdf]

Role of Protonation States in Stability of Molecular Dynamics Simulations of High-Resolution Membrane Protein Structures

Jonathan Lasham[1], Amina Djurabekova[1], Volker Zickermann[2,3], Janet Vonck[4], Vivek Sharma[1,5]*

[1]Department of Physics, University of Helsinki, 00014 Helsinki, Finland.

[2]Institute of Biochemistry II, University Hospital, Goethe University, 60590 Frankfurt am Main, Germany.

[3]Centre for Biomolecular Magnetic Resonance, Institute for Biophysical Chemistry, Goethe University, 60438 Frankfurt am Main, Germany.

[4]Department of Structural Biology, Max Planck Institute of Biophysics, 60438 Frankfurt am Main, Germany.

[5]HiLIFE Institute of Biotechnology, University of Helsinki, 00014 Helsinki, Finland.

* correspondence: vivek.sharma@helsinki.fi

Supporting Material

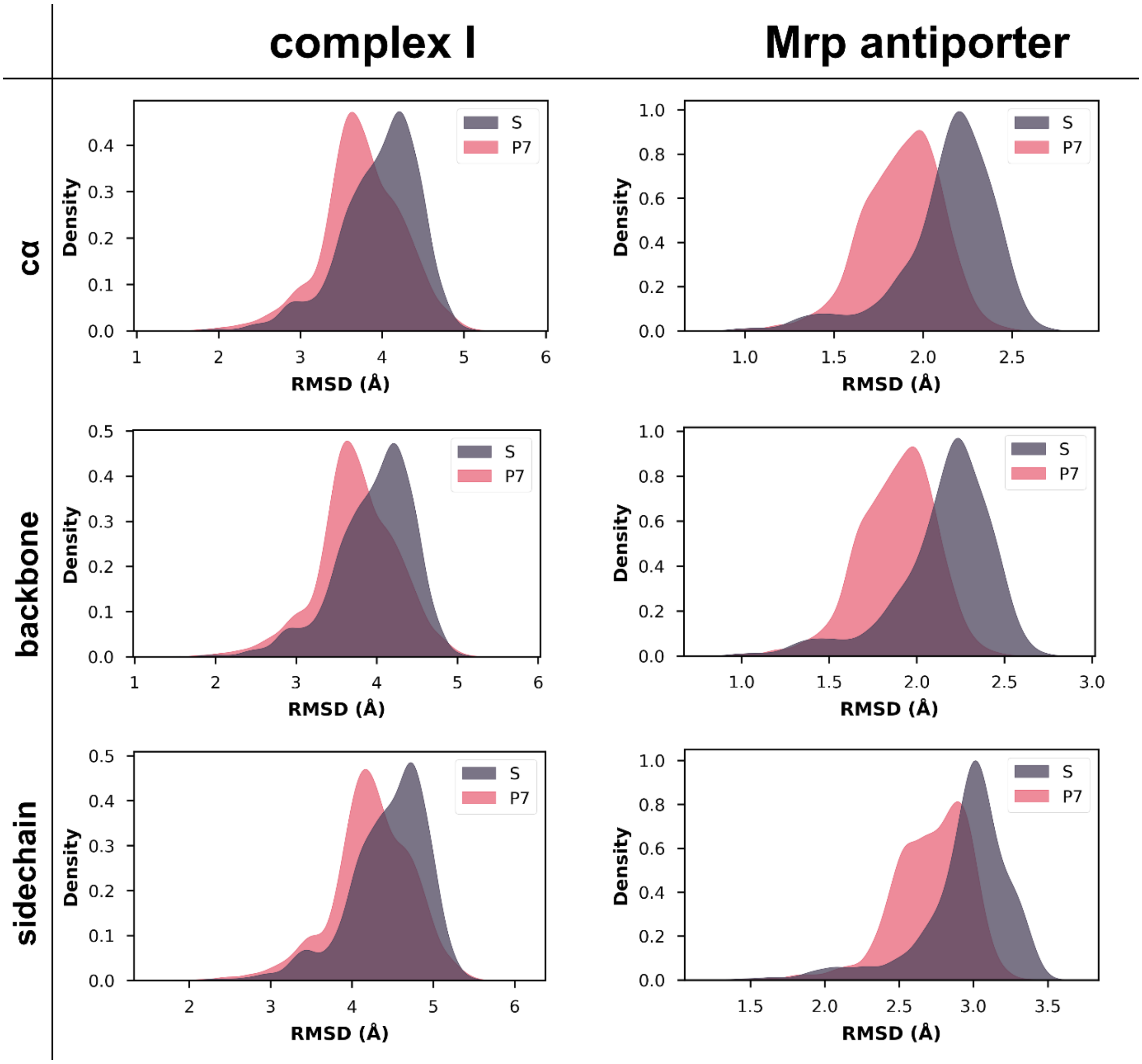

Figure S1 – RMSD (root mean square deviation) over time for both complex I and Mrp systems. The data is combined for all simulations replicas (3 x 1000ns, see methods), and the RMSD is measured every 1 ns. The charts show the distribution of values in the S and P7 states using a kernel density estimate function (KDE) with combined data of all three replicas. Upper panels show the RMSD for Cα atoms, middle panels for backbone atoms, and lower panels for all protein sidechain atoms excluding hydrogens. Atoms in the hydrophilic domain of complex I were included in all plots.

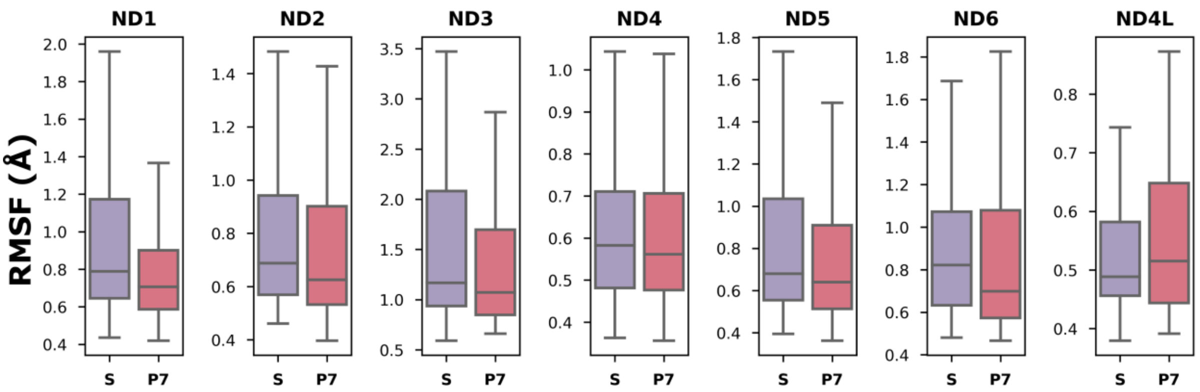

Figure S2 - RMSF (root mean square fluctuation) of Cα atoms of core membrane-bound complex I subunits. The data shown is based all simulation data for S and P7 states (3 x 1 µs, see methods). The shaded box represents the interquartile range, with the middle line showing the median. The upper and lower lines are the maximum and minimum values, respectively.

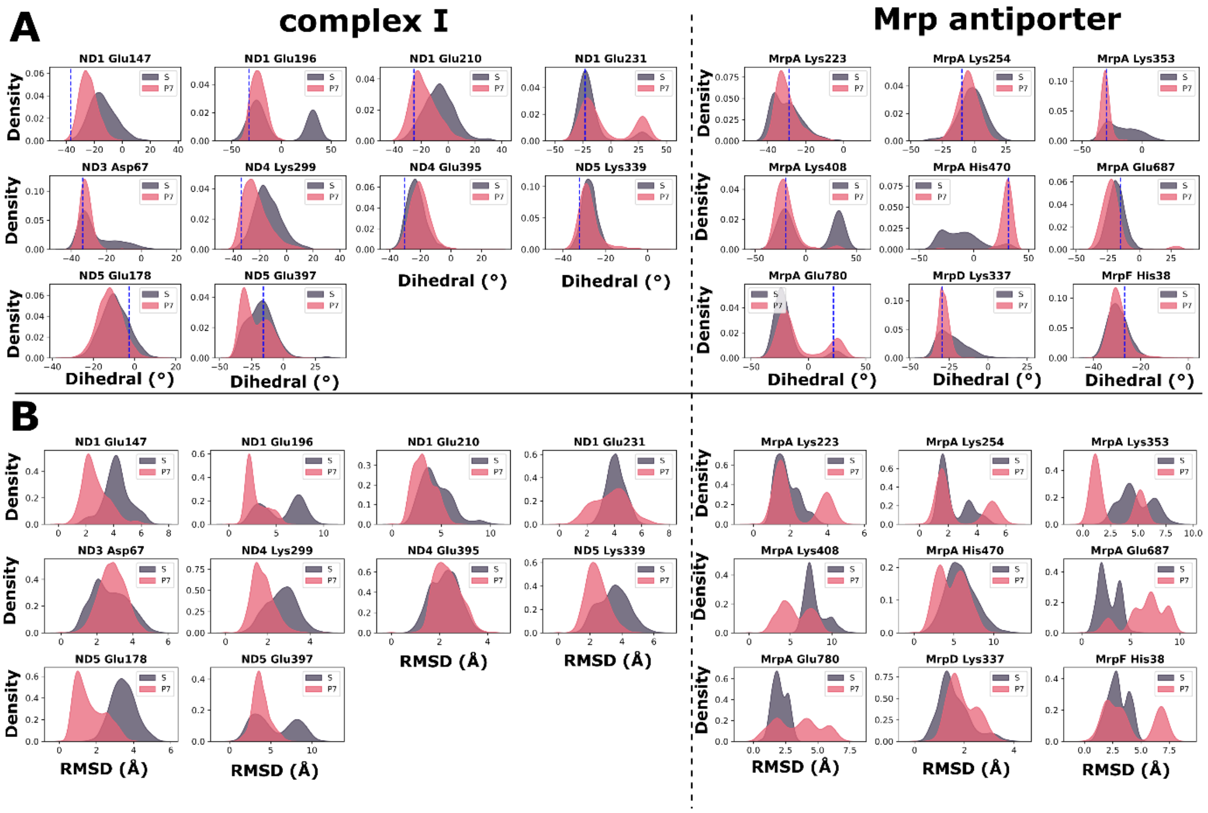

Figure S3 – Analysis of side chain behavior in selected titratable residues that change protonation states between S and P7 states. The panels on the left are for respiratory complex I and on the right for Mrp antiporter. **A** shows the dihedral angle C-CA-CB-CG, with the blue dotted line showing the angular value from the cryo-EM structure. **B** shows the RMSD of the sidechain (excluding hydrogens) with respect to the cryo-EM structure.

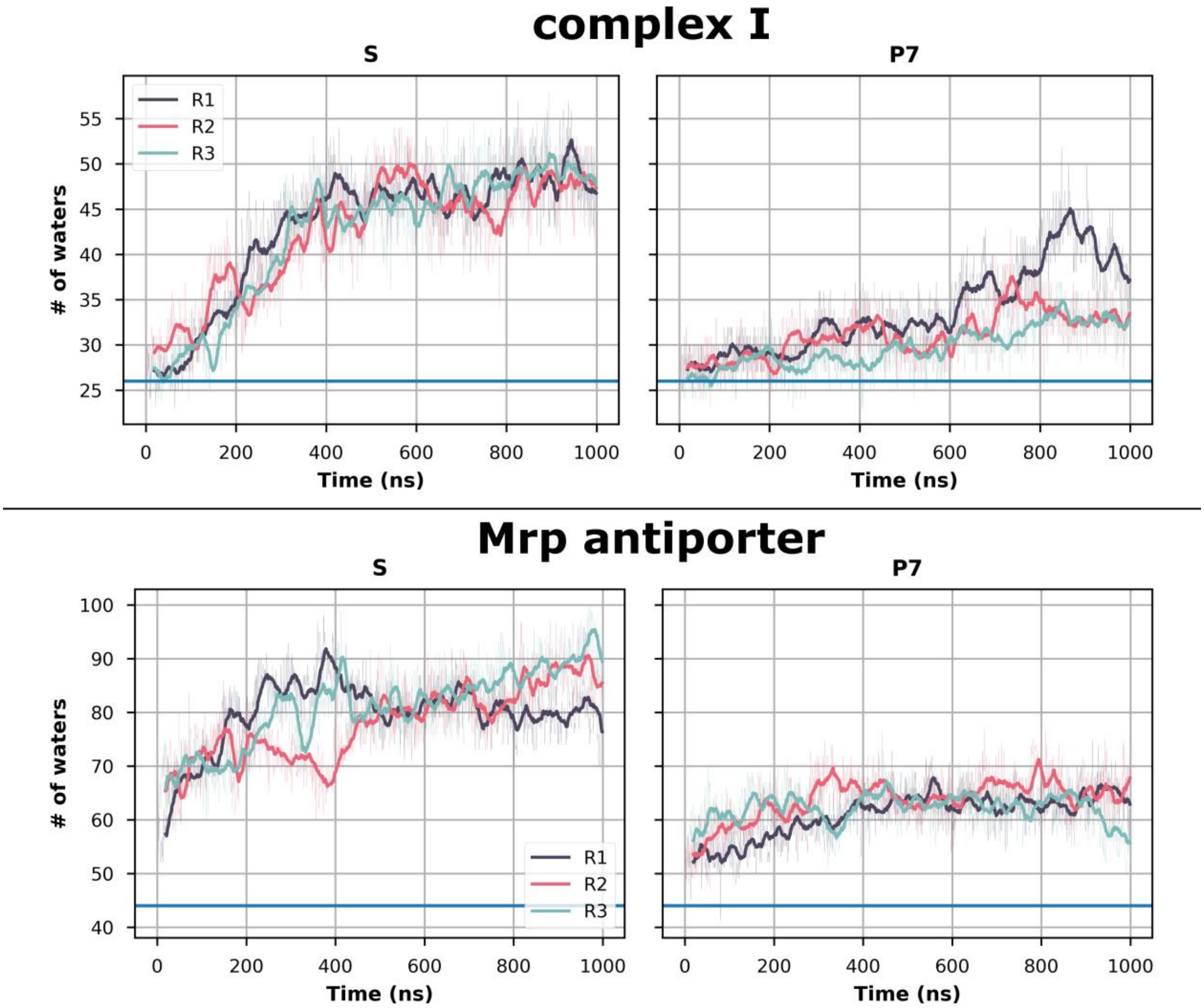

Figure S4 – Time-series of hydration in the hydrophilic central axis of complex I (upper) and Mrp antiporter (lower). Water residues were counted every frame within 4 Å of selected residues. The blue horizontal line represents the number of water molecules seen in the cryo-EM structure.

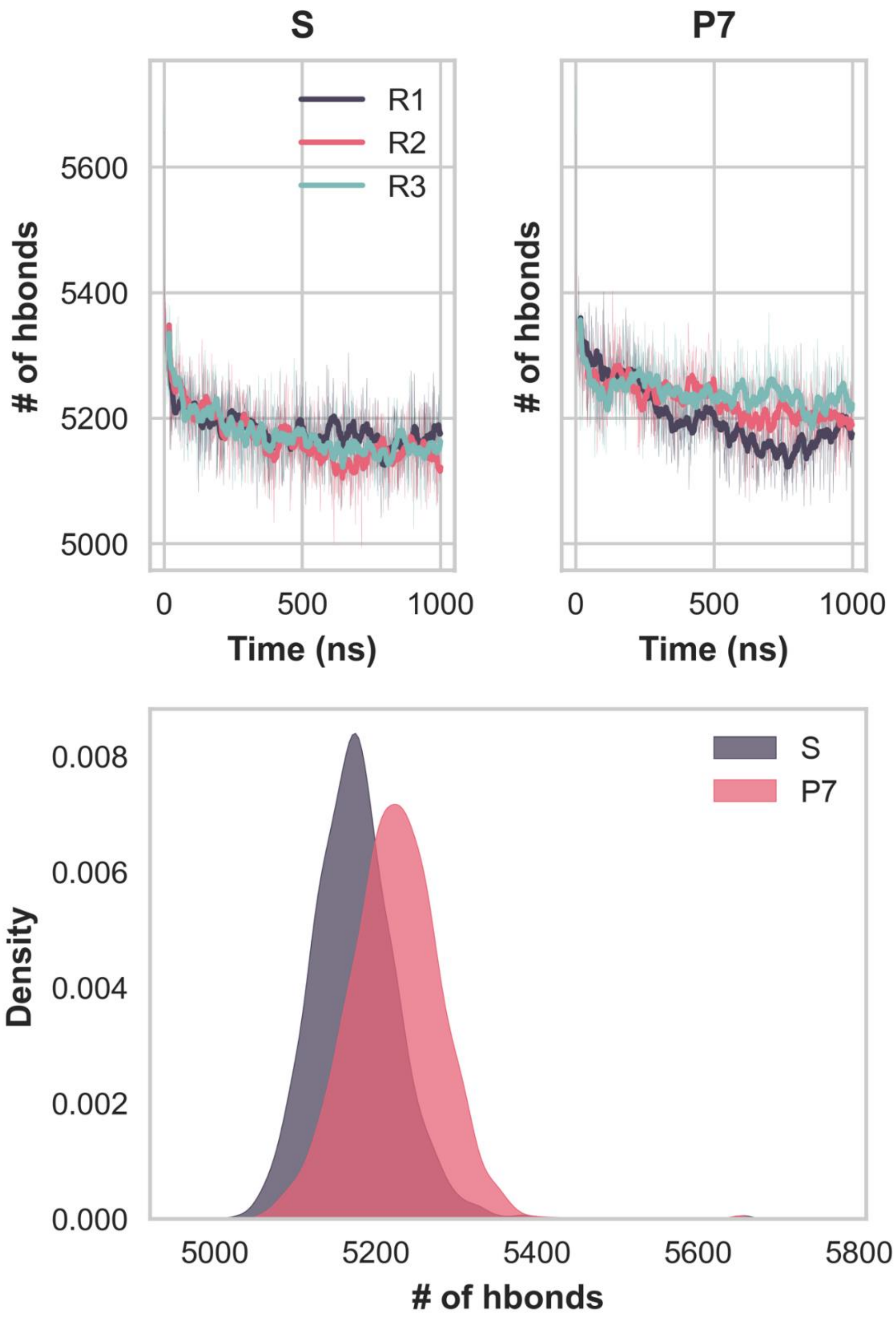

Figure S5 - Number of hydrogen bonds between protein side chains throughout the simulation for all protein subunits in complex I. Top panels show the time evolution of all hydrogen bonds for three different simulation replicas, with the hydrogen bonding distance was cut off at 3.5 Å and the angle at 150̊. Bottom panels show the same data as KDE plots.

Table S1 – List of simulation setups and lengths.

	**State**	**Replicas x length**
**Complex I**	S	3 x 1000ns
	P7	3 x 1000ns
**Mrp antiporter**	S	3 x 1000ns
	P7	3 x 1000ns
	P5	3 x 645ns
	P6	3 x 645ns
	P8	3 x 645ns
	P9	3 x 645ns

Table S2 – Titratable residues in complex I which change protonation state in setup P7. The pKa calculated by PropKa is shown. Only residues in the core membrane subunits are shown.

Subunit	Residue	pKa	P7
ND1	Glu147	11.00	0
	Glu196	9.52	0
	Glu206	8.96	0
	Glu210	7.41	0
	Glu231	7.49	0
	Lys285	6.61	0
ND2	Asp39	7.97	0
	Asp69	8.43	0
	Lys282	6.27	0
ND3	Asp67	7.19	0
	Glu39	7.04	0
	Glu69	9.27	0
ND4	Glu142	8.16	0
	Glu395	7.57	0
	Lys252	6.75	0
	Lys299	6.47	0
ND5	Asp178	9.04	0
	Asp397	7.91	0
	Glu80	7.35	0
	Glu503	8.79	0
	Lys339	6.48	0
	Lys581	6.80	0
ND4L	Asp49	7.20	0
	Glu30	7.60	0
	Glu66	9.77	0

Table S3 – Titratable residues in Mrp antiporter whose charge states were changed in MD setups. The setups P5-9 represent pH values of 5-9. The pKa estimate based on PropKa calculation is shown.

**Subunit**	**Residue**	**pKa**	**P5**	**P6**	**P7**	**P8**	**P9**
MrpA	Asp678	7.36	0	0	0	-1	-1
	Asp771	7.65	0	0	0	-1	-1
	Asp776	5.77	0	-1	-1	-1	-1
	Glu59	6.28	0	0	-1	-1-	-1
	Glu107	5.51	0	-1	-1	-1	-1
	Glu140	6.95	0	0	-1	-1	-1
	Glu157	5.37	0	-1	-1	-1	-1
	Glu366	6.50	0	0	-1	-1	-1
	Glu409	6.79	0	0	-1	-1	-1
	Glu503	5.59	0	-1	-1	-1	-1
	Glu687	7.75	0	0	0	-1	-1
	Glu707	5.90	0	-1	-1	-1	-1
	Glu780	9.52	0	0	0	0	0
	His5	5.13	+1	0	0	0	0
	His60	5.89	+1	0	0	0	0
	His155	5.77	+1	0	0	0	0
	His230	5.84	+1	0	0	0	0
	His365	5.57	+1	0	0	0	0
	His470	7.17	+1	+1	+1	0	0
	His528	5.89	+1	0	0	0	0
	Lys223	6.65	+1	+1	0	0	0
	Lys254	5.90	+1	0	0	0	0
	Lys299	6.84	+1	+1	0	0	0
	Lys353	6.43	+1	+1	0	0	0
	Lys408	6.37	+1	+1	0	0	0
MrpB	Asp121	7.35	0	0	0	-1	-1
	Asp142	5.53	0	-1	-1	-1	-1
	Glu113	6.49	0	0	-1	-1	-1
	Glu141	5.83	0	-1	-1	-1	-1
MrpC	Asp111	5.39	0	-1	-1	-1	-1
	His58	5.3	+1	0	0	0	0
	His98	5.06	+1	0	0	0	0
MrpD	Glu60	5.69	0	-1	-1	-1	-1
	Glu137	6.55	0	0	-1	-1	-1
	Glu467	5.10	0	-1	-1	-1	-1

	His26	5.67	+1	0	0	0	0
	His266	5.50	+1	0	0	0	0
	His366	5.08	+1	0	0	0	0
	Lys161	8.87	+1	+1	+1	+1	0
	Lys219	8.01	+1	+1	+1	+1	0
	Lys250	6.31	+1	+1	0	0	0
	Lys297	7.88	+1	+1	+1	0	0
	Lys337	5.61	+1	0	0	0	0
	Lys392	7.38	+1	+1	+1	0	0
	Lys424	7.05	+1	+1	+1	0	0
MrpE	Asp74	5.32	0	-1	-1	-1	-1
	Glu67	5.72	0	-1	-1	-1	-1
	Glu150	5.77	0	-1	-1	-1	-1
	His146	6.88	+1	+1	0	0	0
MrpF	Asp38	7.04	0	0	0	-1	-1
	Glu56	5.50	0	-1	-1	-1	-1
	Glu62	5.10	0	-1	-1	-1	-1
	Lys80	8.84	+1	+1	+1	+1	0
MrpG	Asp31	5.56	0	-1	-1	-1	-1
	Glu64	5.49	0	-1	-1	-1	-1
	His62	6.25	+1	+1	0	0	0
	Lys41	7.49	+1	+1	+1	0	0

Table S4 – Occupancy of hydrogen bond between structural water and protein sidechain in MD simulations of Mrp antiporter. The subunit names shown in superscript (A/D/F – MrpA/MrpD/MrpF). The RMSF of the residue sidechain is also shown. The data is calculated based on all simulation frames from all trajectories. RMSF is shown as an average of all heavy sidechain atoms. Hydrogen bonds which stabilized more than 20% are shown here. Note complex I had > 90 such hydrogen bonds that stabilized more than 20% occupancy.

	**Hydrogen bond occupancy (%)**			**Sidechain RMSF (Å)**		
	S	P	Change	S	P	Change
**T264^{A}**	11	61	+50	0.83	0.74	-0.09
**N763^{A}**	6	48	+42	0.68	0.66	-0.02
**S479^{A}**	5	41	+36	1.01	0.89	-0.12
**D128^{D}**	8	34	+26	0.92	0.79	-0.13
**Y258^{A}**	0	24	+24	0.70	0.63	-0.04
**E62^{F}**	10	31	+21	1.03	0.96	-0.08

Table S5 – Hydrogen bond occupancy between selected protein side chains in Mrp antiporter. The data is calculated based on all simulation frames from all trajectories. The asterisk (*) denotes residues that were neutralized in the P state. Interactions present in the cryo-EM structure are in green.

	Hydrogen bond occupancy (%)		
	S	P	Change
**$H349^{A}$-$K254^{A}$***	0	76	+76
**$T306^{A}$-$H349^{A}$**	0	66	+66
**$S421^{D}$-$H303^{D}$**	18	82	+64
**$Y447^{A}$-$S305^{A}$**	7	71	+64
**$Y161^{A}$-$D572^{A}$**	1	47	+56
**$K165^{A}$-$D572^{A}$**	12	67	+55
**$S170^{D}$-$E137^{D}$**	32	86	+54
**$H333^{D}$-$K250^{D}$***	0	52	+52
**$R194^{A}$-$E59^{A}$**	32	82	+51
